# Supervised exercise training and increased physical activity to reduce cardiovascular disease risk in women with polycystic ovary syndrome: study protocol for a randomized controlled feasibility trial

**DOI:** 10.1186/s13063-019-3962-7

**Published:** 2020-01-20

**Authors:** Amie Woodward, David Broom, Caroline Dalton, Mostafa Metwally, Markos Klonizakis

**Affiliations:** 1 Centre for Sports and Exercise Science, Faculty of Health and Wellbeing, Sheffield Hallam University, Collegiate Crescent, Sheffield, S10 2BP UK; 2 Biomolecular Research Centre, Faculty of Health and Wellbeing, Sheffield Hallam University, Howard Street, Sheffield, S1 1WB UK; 3 Jessop Wing, Tree Root Walk, Sheffield, S10 2SF UK; 4 Faculty of Health and Wellbeing, Sheffield Hallam University, Collegiate Crescent, Sheffield, S10 2BP UK

**Keywords:** Cardiovascular disease, Exercise, Low-density lipoprotein, Metabolism, Polycystic ovary syndrome

## Abstract

**Background:**

Polycystic ovary syndrome (PCOS) affects up to 20% of women and is characterised by higher amounts of visceral fat, obesity, insulin resistance, dyslipidemia and reproductive and cardiometabolic complications. Increased oxidised low-density lipoprotein (LDL) concentrations have been associated with an increased risk of cardiovascular disease (CVD)-related events. Oxidised LDL is rarely used as a marker for CVD risk in PCOS-related studies despite its widely accepted role in atherogenesis and the increased risk factors associated with PCOS. Additionally, prolonged periods of sedentary behaviour can negatively affect metabolic health. No studies have specifically examined the effects of reducing sedentary behaviour on CVD risk in PCOS with a lifestyle physical activity intervention. The aim of the current study is to measure the feasibility of a randomised controlled trial (RCT) examining the effects of supervised exercise and reducing sedentary behaviour in women with PCOS on CVD risk.

**Methods/design:**

A feasibility, exploratory RCT will be conducted. Fifty-one pre-menopausal females will be randomly allocated between an exercise group (EG), a lifestyle physical activity group (LPAG) and a control group. Participants in the EG will undertake a 12-week supervised aerobic exercise programme. The LPAG will aim to increase daily physical activity and reduce sedentary behaviour for 12 weeks. The control group will not take part in any intervention. Primary outcomes are feasibility and acceptability of the intervention and procedures. Secondary outcomes are oxidised LDL, aerobic fitness, blood lipid profile, fasting glucose and insulin, testosterone and inflammatory markers.

**Discussion:**

PCOS is associated with various increased risk factors for CVD, including hypertension, dyslipidemia, abdominal obesity, insulin resistance, and inflammation. Whether oxidised LDL has a role in this increased risk is not yet known. The present study aims to measure the feasibility of implementing structured exercise training and/or increased lifestyle physical activity in women with PCOS, so that a subsequent adequately powered RCT can be designed. The results from the study will be used to refine the interventions and determine the acceptability of the study design. A limitation is that some self-monitoring in the lifestyle physical activity group may not be reliable or replicable, for example inputting information about time spent cleaning/gardening.

**Trial registration:**

ClinicalTrials.gov, NCT03678714. Registered 20 September 2018.

## Background

Polycystic ovary syndrome (PCOS) is the most common endocrine disorder of reproductive age, affecting 15–20% of women [1]. The diagnostic ‘Rotterdam’ criteria state that women must present with two of the following three signs/symptoms—hyperandrogenism, chronic anovulation/oligomenorrhea, and polycystic ovaries—in the absence of other diseases that promote these symptoms [2]. PCOS is characterised by higher amounts of visceral fat, obesity, and insulin resistance [3] and associated with reproductive and cardiometabolic complications [4].

Dyslipidemia is prevalent in up to 70% of women with PCOS [5]. The lipoprotein profile of women with PCOS is characterised by elevated plasma triglycerides (TG) and reduced high density lipoprotein (HDL) concentrations [6]. Women with PCOS are at increased risk of cardiovascular disease (CVD) compared with weight-matched counterparts. Given the large proportion of lean and overweight women who are affected by PCOS, it is prudent to examine how CVD risk can be mitigated in this high-risk population. Structured exercise has a positive impact on women with PCOS and programmes of 12 to 24 weeks can improve ovulation rates and insulin sensitivity, and aid weight loss [3].

To our knowledge, no studies have examined the impact of lifestyle physical activity and reducing sedentary behaviour on CVD risk in women with PCOS. In addition, oxidised low-density lipoprotein (LDL) is rarely used as a marker for CVD risk in PCOS-related studies despite its widely accepted role in atherogenesis. Increased oxidised LDL concentrations have been associated with an increased risk of CVD-related events and have been shown to accurately predict coronary artery disease [7–9]. Total cholesterol:high-density lipoprotein (TC:HDL) ratio and abdominal obesity are both positively correlated with oxidised LDL [7, 10]. Hence, considering the high prevalence of dyslipidemia and visceral fat in PCOS populations, they may have a higher risk for elevated oxidised LDL concentrations.

Additionally, research pertaining to the cardiometabolic profile in women with PCOS has often produced inconsistent results, possibly due to the wide range of PCOS phenotypes possible once classified by the Rotterdam criteria [11]. Phenotypes that present with hyperandrogenism have been shown to have a worse metabolic profile and increased cardiovascular risk factors than other phenotypes, despite comparable distributions of body weight [11]. Regardless, there are no specific guidelines for this higher risk phenotype.

Finally, recent research has emphasised that even when adults meet physical activity guidelines of 150 min/week [12], prolonged periods of sedentary activity still negatively affect metabolic and general health [13, 14]. High levels of sedentary behaviour promote hyperinsulinemia and subsequently affect insulin sensitivity and glucose concentrations [13], and as such sedentary behaviour may be an exacerbating factor of PCOS.

Before an adequately powered RCT measuring the effects of exercise and/or increased lifestyle physical activity on such indicators of cardiovascular health can be designed, the interventions must be refined and a sample size must be calculated.

Therefore, the specific aims of the present feasibility study are the following:

1. Assess the feasibility of conducting a randomised-controlled trial (RCT) of exercise training and increased physical activity in women with PCOS
2. Identify the use of partitioning participant data by androgen profile
3. Measure rates of recruitment and retention
4. Measure rates of attendance and compliance with the supervised exercise programme
5. Conduct post-intervention interviews with completers and dropouts to refine the interventions and assess barriers to physical activity, and how they can influence future exercise interventions in women with PCOS
6. Obtain a standard deviation for oxidised LDL so that a sample size for a future, larger-scale RCT can be calculated

## Methods/design

### Study design

This study is a feasibility study involving an exploratory RCT. Fifty-one adult females with PCOS who are aged 18 years or over and pre-menopausal will be randomly assigned to a 12-week exercise intervention, an increased lifestyle physical activity group, or a control group. Outcomes will be measured pre- and post- intervention. Interview sessions will be undertaken with a proportion of participants at the end of the trial to provide qualitative information regarding barriers and facilitators to increased physical activity. The study is being conducted at the Centre for Sport and Exercise Science (CSES), Sheffield Hallam University, Sheffield, UK. Sheffield Teaching Hospitals, Sheffield, UK, will be used as a participant recruitment centre.

The study duration is from September 2018 up to May 2021 and is sponsored by CSES, Sheffield Hallam University.

### Recruitment of participants

Participants will initially be screened according to inclusion criteria by a clinical member of the research team via a computerised search of their notes. Afterwards potential participants will be sent information packs about the research, including an invitation letter and participant information brochure. Email and contact information for the researcher will be included in the packs and potential participants will be given a minimum of 24 h and a maximum of 14 days to respond. It will be clearly stated in the invitation letter that there is no obligation or pressure to participate in the study and that if patients do not wish to participate, their future medical care will not be jeopardised.

Volunteers wishing to participate in the study will be requested to contact a member of the research team or respond via letter by a specified date. Participants who respond to the letter of invitation and satisfy triage through telephone pre-screening will be invited into CSES of the Sheffield Hallam University for their initial session, where they will provide written informed consent and have an opportunity to familiarise themselves with the protocol and ask questions. They will then undertake baseline assessments.

It will be assumed that those who haven’t contacted the research team could still potentially have an interest in study participation but may be too busy to respond. They will be re-contacted via telephone (or sent a follow-up letter if telephone contact cannot be established) to remind them of the invitation to the study. Potential participants who decline to volunteer or who do not respond to the follow-up letter will not be contacted further, since it will be assumed that they do not wish to participate in the study.

### Eligibility criteria

Participants eligible for the trial must meet the below inclusion criteria as follows:

i) Women diagnosed with PCOS according to the Rotterdam criteria 2003, National Institute of Health (NIH) 1990 criteria or Androgen Excess and Polycystic Ovary Syndrome (AE-PCOS) Society 2006 criteria
ii) Have experienced menarche (their first menstrual bleeding) and be at least 18 years of age
iii) Are English speaking
iv) Are physically able to perform exercise

Exclusion criteria include:

i) Post-menopausal status
ii) Are smokers
iii) Are undertaking regular structured exercise defined as > 150 min/week
iv) If taking metformin, have been taking it for < 3 months
v) Are taking the oral contraceptive pill (OCP) or have taken it in the last month
vi) Have any medical condition that may be responsible for the symptoms of PCOS, such as congenital hyperplasia, androgen-secreting tumour, hyperprolactinemia, or Cushing’s syndrome
vii) Have current, clinically defined CVD or a history of cardiac events

Participants will be advised that commencement of any of the above-mentioned medications during the trial is a contraindication and they will be withdrawn from the trial.

### Baseline and post-intervention measurements

During visit 1, after written informed consent has been obtained and eligibility confirmed by AW, the following baseline tests measurements will be made: age, anthropometric measures (stature, body mass, hip and waist measurements), finger-prick and venous blood sampling (see Outcome measures for a detailed description of analytes), aerobic fitness assessed by the Astrand-Rhyming single stage test (see Outcome measures for detailed description).

After completion of the intervention, all tests and measurements will be repeated.

### Randomisation and masking

Participants will be randomised in equal numbers between a supervised exercise programme, a lifestyle physical activity group and a control group. This will be undertaken using a computerised randomisation programme (QuickCalcs, GraphPad Software, USA) by an experienced researcher who is not involved in the study assessments. Allocation will be placed in sequentially labelled opaque envelopes. Each envelope will be offered to the participants, in sequence, by the chief investigator, on completion of their baseline assessments. It is not possible to mask participants or research team to the allocated intervention. Outcome assessors will be blinded to group allocation for anthropometric and fitness measures, but blood assays will be carried out by the same member of the research team. This is because blood assays are not considered to be subjective and are unlikely to be affected by outcome assessor bias.

### Withdrawals

Participants may withdraw at any time without providing a reason. They may also choose to withdraw their data from the study. A participant will be considered to be withdrawn if they request to leave the trial or they are lost to follow-up. If allocated to the exercise group, participants will be considered as withdrawn if they no longer attend the supervised exercise sessions, and in the lifestyle physical activity group if they no longer monitor daily physical activity through the fitness application. Data from withdrawn participants will not be used; only data from participants who completed the intervention and baseline and post-intervention assessments will be used in analysis.

### Harms and auditing

Adverse events will be collected, reported and assessed by the research team to determine severity and whether they are likely to be serious adverse events due to the trial. Serious adverse events and reactions will be reported to the relevant ethics committees and appropriate action taken, if any. Auditing will be carried out by the sponsor, Sheffield Hallam University. In the event of harm to participants arising as a result of the management, design or conduct of the research, Sheffield Hallam University insurance and indemnity policies will apply.

### Supervised exercise programme

Participants assigned to the exercise group will be invited to undertake two sessions of supervised exercise training each week for 8 consecutive weeks and three sessions of supervised exercise training each week for the final 4 consecutive weeks at CSES at Sheffield Hallam University. Each session will last approximately 60 min and will involve 40 min of an individualised exercise protocol performed either on a cycle ergometer or a motorized treadmill preceded by a 10 min warm-up and followed by a 10 min cool down.

The duration and intensity of the programme was selected based on evidence from exercise trials in PCOS that have identified that supervised, moderate-intensity exercise sessions between 50 and 70% VO_2max_ for a minimum of 12 weeks showed improvement in cardiometabolic risk factors such as TG, inflammation, and insulin resistance [3, 4, 15, 16].

Most of these studies involved three sessions per week [3, 15, 16]. However, to maximise adherence and reduce inconvenience to participants, the current trial comprises two sessions per week, at a duration of 1 h, increasing to three sessions per week for the final 4 weeks. Various research studies have shown that two sessions per week for 8 weeks can elicit improvements in microvascular endothelial function and exercise tolerance, both of which can reduce cardiovascular disease risk [17, 18], which is regarded as a key outcome in the current study. Additionally, the American College of Sports Medicine (ACSM) recommends a weekly duration of 60–150 min of exercise duration for inactive individuals or individuals who do not participate in any habitual physical activity [19].

ACSM’s Frequency, Intensity, Time and Type (FITT) principle recommends increasing one variable of the principle after at least one month of exercise. Extremely deconditioned to moderately deconditioned individuals are recommend to work at a moderate intensity of ~ 57–74% HR_max_, which correlates with previous exercise interventions in PCOS where workload is set to a VO_2max_ of 50–70% [19]. The programme therefore increases in intensity at increments of 4 weeks up to 74% HR_max_, allowing for progression while remaining moderate in effort and within the recommendations for moderately deconditioned individuals.

In order to calculate individual heart rate zones for each participant, a formula (206.9 − (0.67 × age)) will be used to calculate maximum heart rate [19]. Participants will wear a Polar T31 heart rate monitor chest strap and wristwatch for the duration of each session in order to monitor their heart rate and stay in the assigned zones.

### Lifestyle physical activity group

Participants randomized to the lifestyle physical activity group will attend CSES at Sheffield Hallam University for all the tests and measurements but will not take part in the structured exercise intervention. However, advice and information on how to increase physical activity will be provided using British Heart Foundation guidelines: ‘Get active, stay active’. This will be discussed during the informed consent consultation. Participants will be asked to monitor and track their daily physical activity using a smartphone fitness application. This will either be Google Fit or Apple Health, since these are the default fitness apps for Android and Apple phones and are often pre-installed. The research team will gain permission to access their recorded activity. Between the baseline measurements and those after 12 weeks, participants will receive regular telephone support, approximately once a week, to obtain information about their progress and provide advice as needed.

Participants in the control group will not undertake any intervention but will still receive standard care from their medical professional which may include general weight-loss advice. To improve adherence for the control group, the research team will offer all participants allocated to the group the opportunity to undertake supervised exercise sessions at the CSES after their successful completion of the trial.

### Participant timeline

**Fig. 1:**
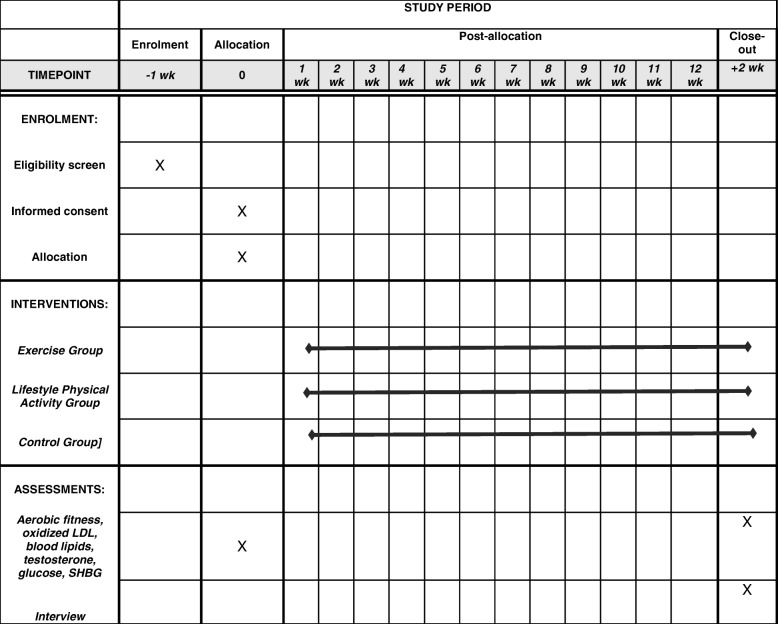
Participant schedule of enrolment, interventions and assessments

The Standard Protocol Items: Recommendations for Interventional Trials (SPIRIT) (Additional file 1) diagram shown in Fig. 1 outlines the participant schedule of enrolment, intervention, close-out and assessment time-points.

### Outcome measures

#### Feasibility outcomes

The primary outcomes for this study are acceptability and feasibility of procedures for recruitment, allocation, measurement, and retention for the intervention procedures. Recruitment rate will be calculated by dividing the number of women eligible and consenting by the recruitment period. Attrition rates will be established as discontinuation of the intervention and loss to follow-up measurement for both conditions. Compliance will be monitored by session attendance and monitoring the data from recorded daily physical activity, with examination of reasons for drop-out or non-compliance. Reasons for drop-out will also be used to assess the suitability of allocation procedures. Suitability of measurement procedures will be evaluated by completion rates and reasons for missing data. The post-intervention interview will be used to assess the acceptability of the exercise programme and lifestyle physical activity group intervention, along with attendance and compliance data.

#### Secondary outcomes

Serum oxidised LDL is the secondary outcome of the current study and will be analysed by enzyme-linked immunosorbent assay (ELISA). Optical density will be measured using an electronic plate reader and can be used to determine the quantity of the specific antigen in the blood using a calibration curve.

ELISA will also be used for quantitative analysis of C-reactive protein, neopterin, TBARS, fasting insulin and sex hormone binding globulin (SHBG). Free testosterone will be measured using a liquid chromatography method. This method has been chosen due to its ability to detect small amounts of serum testosterone, particularly in lower concentrations in females [20]. Free testosterone will be quantified using a calibration curve and calculating peak area.

Aerobic fitness will be assessed using the Astrand-Rhyming test. This is a submaximal single-stage test performed on a cycle ergometer, lasting between 6 and 7 min [21]. The goal is to obtain heart-rate values between 125 and 170 beats per minute (bpm) for a given work-rate at 50 revs/minute (50 or 75 watts for unconditioned women) [21]. Heart rate is then measured at the fifth and sixth minute if steady state (a difference ≤ 5 bpm is achieved), and the average of the two heart-rate measurements can be used to estimate VO_2max_ according to a nomogram. This value must be adjusted for age to account for decreasing maximal oxygen uptake and maximal heart rate with age [19].

For lipid profile (including LDL-cholesterol, HDL-cholesterol, TG and TC) and fasting glucose, a Cholestech Automated Analyser will be used. A fingerprick blood sample is obtained and collected into a capillary tube. The sample is then inserted into the machine cassette. This produces an automated reading from the sample in which accuracy can be maintained by using control samples and calibrations. HDL sub-fractions will be measured using gel electrophoresis.

In order to measure the amount of lifestyle physical activity (and subsequent sedentary activity), the long-form International Physical Activity Questionnaire (IPAQ) will be administered at baseline and post-intervention to all participants. This will be used to compare differences in time spent sitting, both during the week and weekend, and the amount of lifestyle physical activity (including transport, housework, and leisure time) undertaken.

Waist circumference will be measured with the participant standing with feet together, and the tape measure placed around the narrowest part of the torso, between the umbilicus and the xyphoid process [19]. Hip circumference will be measured with the participant standing as above, and the tape measure placed around the maximum circumference of the buttocks [19].

### Blood sampling and storage

Blood will be drawn from participants on their initial and post-intervention visit to the CSES, by a member of the research team trained in venepuncture. Blood samples will be centrifuged and serum aliquoted and stored at − 80 °C until they are analysed.

Assays will be performed at the Biomolecular Research Centre, Sheffield Hallam University, by a member of the research team.

### Data collection, monitoring, management and storage

Data will be collected by the chief investigator (AW) using data collection forms and entered into a computerised database. Range checks will be carried out to ensure data quality. Data checks will frequently be completed by other members of the research team.

Participants’ names will be anonymised and replaced with a code using a computerised pseudonymisation programme (Open Pseudonymiser, University of Nottingham, UK). All other study data will be stored securely on Sheffield Hallam University premises and/or saved on encrypted computer drives on site. Only the research team will have access to the data, including the final trial dataset. Data will be securely archived after study closure and stored for up to 7 years, when it will be destroyed. All data will be stored and managed according to Sheffield Hallam University’s confidentiality and data protection policies.

Due to the low risk involved in this study, no formal data monitoring committee will be formed. However, the study will be regularly monitored by the research team members being led by a senior team member (MK) who will meet at regular intervals throughout the study period.

### Interview and qualitative methods

After trial completion, four participants from each group, as well as drop-outs, will be offered the opportunity to complete a semi-structured interview either in person or over the telephone with a member of the research team. All interviews will be recorded with an audio recording device and then transcribed verbatim. The interview will explore their opinions and experiences of the intervention, and factors that may be a barrier or facilitator to physical activity in the future. It will also assess the acceptability of the exercise intervention and study procedures, to refine the design and delivery of the exercise programme for the RCT, and will last approximately 45 min.

Interviews will allow the exploration of participant’s motivations, attitudes and experience in detail and within their personal frame of reference. This will be useful because of the wide-ranging symptoms of PCOS, which may affect individuals in a variety of ways that are not necessarily uniform across all participants. Transcripts will be analysed using thematic analysis [22]. Thematic analysis is an inductive method for identifying, analysing and reporting patterns within data. As such, it allows for a great deal of flexibility in analysing qualitative data because it is independent of theory and epistemology; rather, it can be applied across a range of theoretical approaches [22]. This allows for themes and patterns to be identified by the researcher without trying to fit it into a pre-existing coding frame, and is therefore considered to be data-driven and accessible [22].

### Data analysis

All quantitative measurements will be presented as mean ± standard deviation (SD) unless otherwise stated.

As this is a feasibility study, no formal sample size calculation was required. The sample size for a feasibility study needs to strike a balance where it does not cause undue burden on participants by being too large, but not be too small that critical parameters (such as consent rate, attrition, and compliance) cannot be precisely estimated and used for the calculation of a full-scale RCT. It has been posited that a rule of thumb of at least 30 participants overall should be sufficient for a feasibility trial [23].

Additionally, sample sizes between 24 and 50 have been recommended to calculate a standard deviation of an outcome that can then be entered into a formal power calculation for the full-scale RCT [24, 25]. Oxidised LDL concentrations are a secondary outcome for the proposed study, and a sample size within this remit will provide a reliable standard deviation for oxidised LDL to be used in a power calculation. Since there are three groups, a total sample size of 51 will allow even numbers across three groups (17 in each group).

Descriptive statistics will be used to characterise the groups at baseline and post-intervention. Pre and post-intervention means will be recorded for each group. Difference between means will be assessed using two-factor mixed-ANOVAs where the effect of two independent variables (grouping and time point) will be measured on the dependent variables. The within-subjects factor is time, and the between-subjects factor is group. Post-hoc analysis will be undertaken in the event of any significant differences in group means. Data will be assessed to ensure the assumptions of the mixed-ANOVA are met including checks for normality.

Using free-testosterone data, all participants will be categorised by androgen profile as normo-androgenic or hyper-androgenic based on laboratory cut-off values [26]. Androgen profile will then be added into the mixed-ANOVA model, where time and androgen profile will be the within-subjects factors and group will be the between-subject factor. Partial eta squared will be presented. Assuming normality, Pearson correlation coefficient will be used to analyse the bivariate correlation between variables. Results of all analyses will be interpreted with a caveat that the study is a feasibility trial without a formal sample size calculation and as such may be inadequately powered. All statistical analysis will be undertaken using the latest IBM SPSS Statistics software, currently version 24.0.

### Dissemination

Trial participants will be informed of the results of the study via a summary report. The results will be disseminated at conferences and through journal publications. Relevant UK PCOS charities will be informed of results in laymen’s terms. No later than 3 years after study closure, an anonymised participant-level dataset will be deposited in an appropriate data archive for sharing purposes. Neither the results of the study nor any information provided by participants or the sponsor for study purposes will be passed on to any third party without consent from the participants and sponsor.

## Discussion

Oxidised LDL has been associated with all stages of atherosclerosis, as well as other conditions linked to CVD, such as diabetes mellitus and metabolic syndrome [27]. PCOS is associated with various increased risk factors for CVD, including hypertension, dyslipidemia, abdominal obesity, insulin resistance, inflammation, and endothelial dysfunction [3, 6]. Whether oxidised LDL has a role in this increased risk in PCOS is not yet known.

The present study aims to measure the feasibility of implementing exercise training and/or a lifestyle physical activity intervention in women with PCOS. This will provide necessary information to design an adequately powered RCT examining the effect of such interventions on oxidised LDL and CVD risk. The strengths of this study include the lifestyle physical activity arm, which will encourage participants to be more active whilst reducing time spent sedentary, and monitor their daily physical activity, so that the effects of this can be compared with a formal, structured exercise programme. Additionally, partitioning data by androgen profile will highlight differences in normo- and hyper-androgenic cardiometabolic profiles and how exercise and physical activity can impact these. This study aims to provide feasibility data and effect sizes in order to plan for a full-scale RCT. In addition, it aims to contribute to recommendations that move away from a one-size fits all approach to PCOS management and move to recommendations based on individual presentations of the condition. A limitation is that some self-monitoring in the lifestyle physical activity group may not be reliable or replicable, for example inputting information about time spent cleaning/gardening.

## Trial status

Protocol version 6.0; 6 September 2018. Recruitment beginning October 2018 until required numbers are reached.

## Supplementary information

**Additional file 1.** Populated SPIRIT checklist.

## Data Availability

Not applicable.

## Abbreviations

ACSM: American College of Sports Medicine
ANOVA: Analysis of variance
CSES: Centre for Sport and Exercise Science
CVD: Cardiovascular disease
ELISA: Enzyme-linked immunosorbent assay
HDL: High-density lipoprotein
HRA: Health Research Authority
LDL: Low-density lipoprotein
OCP: Oral contraceptive pill
PCOS: Polycystic ovary syndrome
RCT: Randomised controlled trial
REC: Research Ethics Committee
SHBG: Sex hormone binding globulin
TBARS: Thiobarbituric acid substances
TC: Total cholesterol
TG: Triglycerides
